# Short tandem repeats in the inhibitory domain of the mineralocorticoid receptor: prediction of a β-solenoid structure

**DOI:** 10.1186/1472-6807-13-17

**Published:** 2013-10-02

**Authors:** Metaxia Vlassi, Katharina Brauns, Miguel A Andrade-Navarro

**Affiliations:** 1 Protein Structure & Molecular Modeling laboratory, Institute of Biosciences & Applications, National Centre for Scientific Research “Demokritos”, 15310 Ag. Paraskevi, Athens, Greece; 2 Max Delbrück Center for Molecular Medicine, Robert-Rössle-Str. 10, 13125 Berlin, Germany

**Keywords:** Mineralocorticoid receptor, Tandem repeats, Protein structure prediction, Molecular dynamics simulation, β-solenoid

## Abstract

**Background:**

The human mineralocorticoid receptor (MR) is one of the main components of the renin-angiotensin-aldosterone system (RAAS), the system that regulates the body exchange of water and sodium. The evolutionary origins of this protein predate those of renin and the RAAS; accordingly it has other roles, which are being characterized. The MR has two trans-activating ligand independent domains and one inhibitory domain (ID), which modulates the activity of the former. The structure of the ID is currently unknown.

**Results:**

Here we report that the ID contains at least 15 tandem repeats of around 10 amino acids, which we computationally characterize in the human MR and in selected orthologs. This ensemble of repeats seems to have emerged around 450 million years ago, after the divergence of the MR from its close homolog, the glucocorticoid receptor, which does not possess the repeats. The region would have quickly expanded by successive duplication of the repeats stabilizing at its length in human MR shortly after divergence of tetrapoda from bony fishes 400 million years ago. Structural predictions, in combination with molecular dynamics simulations suggest that the repeat ensemble forms a β-solenoid, namely a β-helical fold with a polar core, stabilized by hydrogen-bonded ladders of polar residues. Our 3D-model, in conjunction with previous experimental data, implies a role of the β-helical fold as a scaffold for multiple intra-and inter-molecular interactions and that these interactions are modulated via phosphorylation-dependent conformational changes.

**Conclusions:**

We, thus, propose that the structure of the repeat ensemble plays an important role in the coordination and sequential interactions of various MR partners and therefore in the functionality and specificity of MR.

## Background

The mineralocorticoid receptor (MR) belongs to the steroid hormone receptor (SHR) subfamily of nuclear receptors. It plays a major role in the regulation of sodium and water homeostasis in epithelial cells of the colon and distal nephron of the kidney as part of the renin-angiotensin-aldosterone system (RAAS). The MR derived from a common ancestor with the glucocorticoid receptor (GR) through duplication at least 450 million years ago [1], predating the emergence of the RAAS and suggesting that the MR has other more ancestral functions [2]. Accordingly, in recent years, additional functions of the MR in cardiovascular regulation, neuronal fate and adipocyte differentiation have been discovered (see e.g. [3] and references therein).

**Figure 1:**
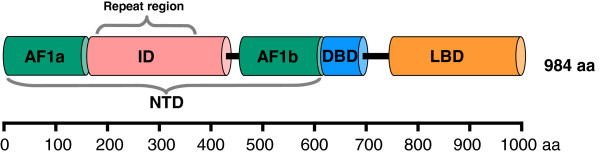
**Domain structure of the human MR.** The N-terminal domain (NTD) is composed of two activatory domains (AF1a and AF1b) and the inhibitory domain (ID). This is followed by the DNA binding domain (DBD) and the ligand (sterol) binding domain (LBD). We found a region with tandem repeats that covers most of the ID starting at its N-terminal. Other isoforms of the human MR have been defined but their splicing does not affect the tandem repeat region [4]. Isoform 1 is displayed (which is the longest one).

The MR is organized into three major protein regions (Figure 1; [4]): the N-terminal domain (NTD: 1-602 aa), the DNA-binding domain (DBD: 603-676 aa), which binds to DNA-sequences on target genes, and the ligand-binding domain (LBD: 737-984 aa) in the C-terminal, which binds to the steroid hormones aldosterone, cortisol and corticosterone. DBD and LBD are connected by a hinge-region (677-732 aa).

The NTD contains two trans-activating ligand independent domains AF1a (1-167 aa) and AF1b (446-602 aa), and one inhibitory domain (ID: 168-445 aa) [4]. The MR recruits through its functional domains (AFs or ID) distinct co-activator or co-repressor complexes to ensure, at the post-receptor level, transcriptional selectivity. The ID is sufficient to limit the activity of the NTD when fused to either of the trans-activating domains [4].

Whereas the structures of LBD and DBD are already known, the folding of the NTD remains to be defined. The NTD is of particular interest because it is specific in sequence to the MR and therefore possibly explains the particular functional variability that distinguishes the MR from other steroid binding receptors. To complete the structural picture of the MR we carried out a sequence similarity analysis of the NTD. A sequence similarity search of the sequence of the human MR against the protein sequence database suggested the existence of tandem repetitive sequences evidenced by multiple partial sequence matches between the repeats in orthologs of the MR across several species.

Tandem repeats form structural ensembles with peculiar characteristics. Their characterization allows the prediction of secondary and tertiary structure and may be useful to suggest particular functions to specific protein regions [5]. To facilitate the study of the structure and function of the NTD of the MR, here we present computational analyses of tandem repeats in the NTD, to describe their evolution and predict their structure. Our results suggest that these repeats fold together to form a β-solenoid domain involved in intra-and inter-molecular interactions.

## Results

### Definition of the repeat ensemble

Sequence similarity search of the sequence of the human MR against the protein sequence database (using BLAST [6]; see Methods for details) indicated multiple matches of similarity between fragments of the human MR and its orthologs in several species evidencing a repetitive pattern within a region of the protein of about 200 amino acids. We followed up this discovery with an iterative procedure where we first aligned a selection of orthologs of the human MR, examined visually patterns of conservation in the corresponding region, and generated increasingly complex regular expressions that were used to search the aligned sequences (using Jalview [7]; see Methods for details) attempting to capture this pattern while minimizing matches outside the repeat region. The multiple sequence alignment was manually modified in some positions to align equivalent matches to the regular expression. Homology of this region was restricted to actinoperygii species (including both bony fishes and tetrapoda), which suggests that the NTD emerged with the MR, and signifies the importance of the NTD as characteristic of the functionality of the MR.

**Figure 2:**
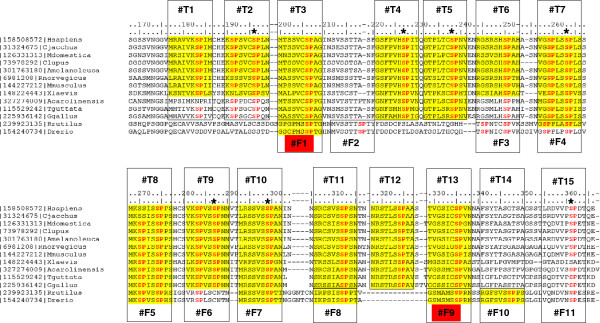
**Multiple sequence alignment of the tandem repeat region.** The positions of tandem repeats of length 10 aa are indicated with boxes in a multiple sequence alignment of the region with the repeats from the human MR with selected orthologs from tetrapoda and two bony fishes (Rutilus and zebrafish, *Danio rerio*) (see Methods for details). For some repeats, alignment between the tetrapoda and the fish repeat might not reflect a direct evolutionary relation but the similar amino acid properties of the repeats; this is indicated with separate boxes for fish repeats. Fish repeats F1 and F9 (red labels) have 6/10 identical positions, suggesting a recent event of repeat duplication in bony fishes. Matches to a regular expression used to identify the repeats ([GIKLMNRSTV] [ACEGNKPRSTV] [ACFGLMNPS] [AIKLPRSTV] [ADGILMPRSTV] [ACGHKRS] S P [AGHILMNPRSTV] [AGHIMNSTV]) are marked in yellow (see Methods for details). Tetrapoda repeats are labeled from #T1 to #T15. Fish repeats are labeled from #F1 to #F11. The region starts at the N-terminal of the ID. The last two repeats were identified with a complementary analysis (See text and Figure 3). The sequence identifiers indicate the Entrez Protein identifier and the species name for human (*Homo sapiens*), common marmoset (*Callithrix jacchus*), gray short-tailed opossum (*Monodelphis domestica*), dog (*Canis lupus familiaris*), panda (*Ailuropoda melanoleuca*), rat (*Rattus norvegicus*), mouse (*Mus musculus*), African clawed frog (*Xenopus laevis*), Carolina anole (a lizard, *Anolis carolinensis*), Zebra Finch (a bird, *Taeniopygia guttata*), chicken (*Gallus gallus*), the common roach (a fish, *Rutilus Rutilus*), and the zebrafish (*Danio rerio*). Known phosphorylation sites [8,9] are indicated by an asterisk (see text for details).

The final regular expression and its matches in a multiple sequence alignment of representative orthologs of the human MR (see Methods for details) suggest that the tetrapoda sequences have at least 13 tandem repeats whereas the bony fish sequences have 10 tandem repeats (Figure 2). The repeats have a length of 10 amino acids with a highly conserved Ser-Pro motif at positions 7 to 8. Linkers between the repeats tend to be very short: for the human MR there is no gap between repeats #2 and #3, but 2 to 3 amino acids is the most common linker length.

We did not observe this tandem repeat in the closest human paralog, the glucocorticoid receptor (GR), or in less related human receptors (Androgen receptor, AR; Progesterone receptor, PR), all of which share significant similarity to the DBD and LBD domains of the MR, which indicates their common evolutionary origin.

The specific presence of the tandem repeats in the MR indicates that their function is specific to this receptor. The increase in the number of repeats between bony fish and tetrapoda suggests that there was evolutionary pressure to increase the length of the ensemble of repeats once they were incorporated in an ancestral version of the MR. This probably occurred in a short (in geological terms) time period, that is, between the divergence of the MR from the GR around 450 million years ago and the divergence of tetrapoda from bony fishes around 400 million years ago. Such variability in an overall large number of elements of an ensemble of tandem repeats suggests that they assemble in an elongated domain as opposed to repeats forming closed structures such as WD40 or Kelch, which tend to appear in multiples of six or seven units [10].

In order to search for instances of these repeats in families distant to the MR, we built a Hidden Markov Model (HMM) of an alignment of the repeats, which was used to search the protein database (using the HHMER web server [11]; see Methods for details). We did not find significant new hits, which supports that these repeats are unique to the MR.

Finding the appropriate frame can be a problem when dealing with tandem repeats. The production of the regular expression was incremental, starting from the conserved Ser-Pro motif, and therefore we could have misidentified the boundaries of the repeat unit; these can be deduced from sequence analysis of the pattern of insertions (which will tend to occur in the linker between repeats), or if a well defined repeat is either followed or preceded by a region clearly not being a repeat.

As a means to complement our analysis we took advantage of several computational methods that are available as web tools for the detection of repeats in protein sequences. An overview of the results obtained with the application of these methods to the human MR is indicated in Additional file 1: Table S1. The Ser-Pro motif was readily detected by some of the tools, which reproduced the frame that we deduced.

### Secondary structure prediction

After defining the sequence of the repeat, we tried to predict its structure. Initially, we approached the prediction of the secondary structure of the repeat region using Jpred3 [12], a tool that, like most secondary structure predictors, takes advantage of the conservation patterns in a multiple sequence alignment (MSA) to aid its predictions. Intriguingly, neither a default analysis of the region nor analyses on manually curated alignments of either one or two consecutive repeats resulted in any predicted secondary structure. This result might be not surprising considering that secondary structure predictors such as Jpred3 are trained and specialized in globular proteins and might not work for isolated short sequences and regions of compositional bias.

Next, we tried a computational tool that predicts contact-dependent secondary structure propensity (CSSP), based on the observation that the conformational preferences of a short sequence are influenced by the context of a native protein scaffold (See [13] and references therein). This method has been successfully used to detect hidden β-propensities and offers the additional advantage of accurate structure prediction even for extremely short sequences. We hypothesized that such an approach could be applied to predict the conformational preferences of the short tandem MR repeats.

**Figure 3:**
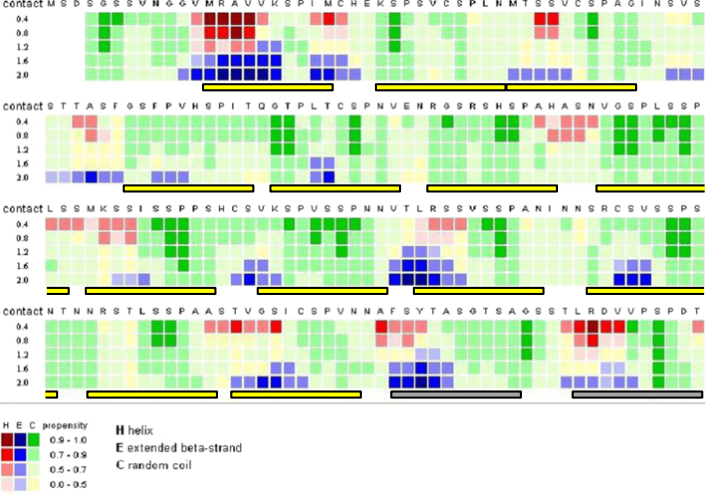
**Contact-dependent secondary structure prediction of the repeat region in human MR.** The CSSP profile [13] of the human MR tandem repeats (165-364 aa) using tertiary contacts (TC) in the range of 0.4 to 2.0 is shown. Predictions for α-helix, β-strand and random coil are colored red, blue and green, respectively, with variable propensity values, as indicated at the bottom of the figure. The position of predicted repeats is indicated under the alignment with boxed bars. Short β-strands are predicted upstream the conserved Ser-Pro motif of each repeat. This analysis confirmed the repeats identified in the pattern analysis (Figure 2) and suggested two additional (very divergent in sequence) repeats, marked with yellow and gray bars, respectively.

As shown by the CSSP profile of this domain (Figure 3), a short region upstream the Ser-Pro motif of each MR repeat, has a relatively high propensity to form a β-strand (in blue in Figure 3). More importantly, the CSSP prediction reflected the periodicity of the repeats and even suggested the presence of two extra repeats (indicated with grey bars in Figure 3), which we had not detected previously due to their extreme sequence divergence (see T14-T15 and F10-F11 in Figure 2). According to the CSSP prediction, the short β-strand is the only structural element in each MR repeat. This prediction is in line with published circular dichroism (CD) data from a fragment of the MR repeat region (MD: aa 247-385), showing a relatively high content of β-strand (22%) and β-turn (24%) structures for this domain, in the absence of structure stabilizing agents (see Table two in [14]: MD buffer). Furthermore, the repetitive structure predicted by CSSP suggests a possible assembly between consecutive MR repeats, where the short β-strands of each of the repeats pack against each other to form β-sheets.

### Evolution of the repeat ensemble

The similarity between *D. rerio* F9 and F1 (highlighted in red in Figure 2) is outstanding: these repeats have 6 out of 10 identical positions (GS--MSSP), the highest level of identity among non-equivalent repeats in the dataset. This reflects a possible event of repeat duplication unique to bony fishes, suggesting evolutionary pressure to increase the size of the ensemble both in the tetrapoda and in the bony fish lineages.

**Figure 4:**
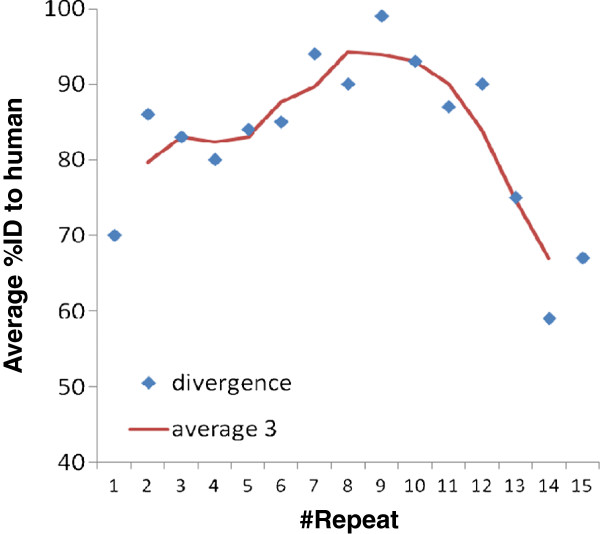
**Similarity between tetrapoda repeats.** The average percentage of identity to each human repeat (T1 to T15) from the corresponding repeats of the other tetrapoda included in the MSA shown in Figure 2 is plotted versus the position of the repeat. Higher values are observed for the middle repeats. The trend line (red) is an average of three consecutive repeats. The plot suggests higher conservation of the middle repeats.

The average identity between tetrapoda repeats to the equivalent human repeat shows that the repeats situated in the middle of the ensemble are more conserved (Figure 4); this suggests that these repeats fold together serially forming an elongated domain where the middle repeats form a core that is more conserved than the repeats at its boundaries.

### 3D-structure prediction

Our CSSP predictions, in conjunction with published CD data on the MD fragment of the MR repeats in buffer (see above) suggested a 3D-fold formed by repeated structural units comprising short β-strands connected by turns, which is reminiscent of β-solenoids [15]. Adding to this observation, the short length of the MR repeats (10 residues) reinforces the idea of a β-solenoid fold. Namely, it has been shown that the solenoid folds are predominant in proteins with repeats of 5 to 40 residues, with β-solenoids corresponding to proteins with the shorter repeats, as they require fewer residues to complete one coil of the solenoid superhelical fold [15,16].

**Figure 5:**
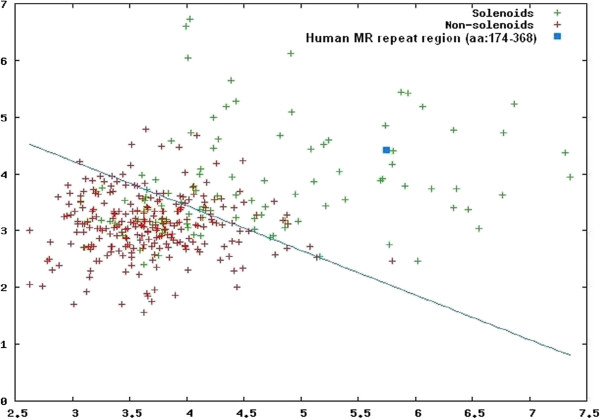
**Output of the REPETITA method.** Scatter plot of optimal θ-ratio versus Z_max_ for the training sets and the human MR repeat region (aa: 174-368). These parameters reflect the existence of a periodic signal in several amino acid properties along the sequence and the largest spectral amplitude of those, respectively. Red and green crosses correspond to solenoid and non-solenoid proteins, respectively. The result corresponding to the human MR repeats is shown as a blue square.

To test the solenoid hypothesis, we used the REPETITA method, which discriminates solenoid from non-solenoid proteins [17]. As shown in Figure 5 (blue square), the REPETITA output (ρ_θ_ = 4.4, Ζ_max_ = 5.6) of the tandem human MR repeats (aa: 174-368) falls into the region of solenoid proteins, suggesting a solenoid fold for this domain with high certainty, as reflected by the large distance (1.85) from the optimal line separating solenoid from non-solenoid proteins (Figure 5). Similar results were obtained for the mouse and *Danio rerio* MR repeat regions (data not shown).

**Figure 6:**
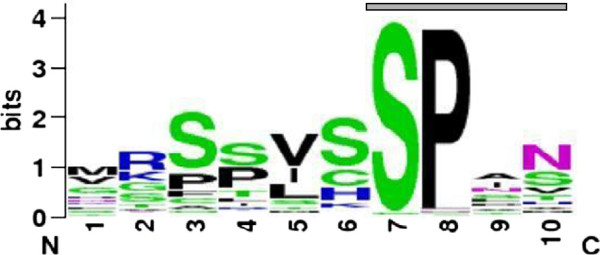
**Sequence logo representation of the MR repeat sequences.** Font color indicates hydrophobic (black), basic (blue), polar (green) and asparagine (magenta) residues. The gray bar indicates the consensus sequence motif, SPxN. The figure was generated with WebLogo [22].

A β-helical fold can also be inferred from two additional observations: first, as detected by a sequence logo of the MR repeat sequences, the MR repeats consistently have aliphatic and polar residues (such as serines and asparagines) at repeat positions 5 and 6, 7, 10, respectively (Figure 6) suggesting a stacking arrangement of these residues. Polar stacks, i.e., hydrogen-bonded ladders of polar side chains and especially the so-called, asparagine ladders, are indicative of right-handed parallel β-helices [18]. Second, according to published data, in the presence of structure-stabilizing agents (trifluoroethanol, TFE), the far-UV CD spectrum of the MD fragment exhibited a deep minimum at ~216 nm, a cross over point near 208 nm and a large maximum at around 197 nm (see Figure four B, in 50% TFE, in [14]), which is reminiscent of the parallel β-helical spectra obtained in the case of the β-helical proteins, PelE and PelC [19]. An additional negative band at 220-230 nm in the spectrum of the MD fragment, however, resembled the signal seen with α-helices [14]. The CD profile of another, highly regular, β-helical protein (antifreeze protein from the beetle, *Tenebrio molitor*) also, superficially, resembled the CD profile typical of an α-helix and this artifact has been attributed to the high regularity of this particular β-helix [20]. Adding to this, other cyclic β-structures also display a CD spectrum typical of an α-helix [21], showing that CD alone can be misleading in the case of some β-helical folds.

Taken together, our observations, so far, support the idea of a regular parallel right-handed β-helical structure for the MR repeats.

### 3D-model of consecutive MR repeats

To further support the hypothesis that the MR repeats fold as a parallel β-helix we generated a detailed model of the 3D-structure of five consecutive MR repeats. First, three consecutive repeats of human MR (T11 to T13) were modeled as a three-coiled right-handed parallel β-helix, using the crystal structure of the *T. molitor* antifreeze protein (PDB code:1EZG, [23]) as template. The choice of this particular template was based on the following similarities with the MR repeats: (i) similar repeat length (12 aa), (ii) the template structure is a right-handed parallel β-helix containing single, short (~3 aa long) β-strands in each β-helical coil, as also predicted by CSSP for the MR repeat region (Figure 3), (iii) the extremely low content of hydrophobic residues (Figure 6) excludes the possibility of a strong hydrophobic core for the MR repeats, as is the case for the template [23] and (iv) the resemblance of the CD profile of the MD fragment of the MR repeats with that of the template (see above) suggests a similar, highly regular β-helical structure [23].

**Figure 7:**
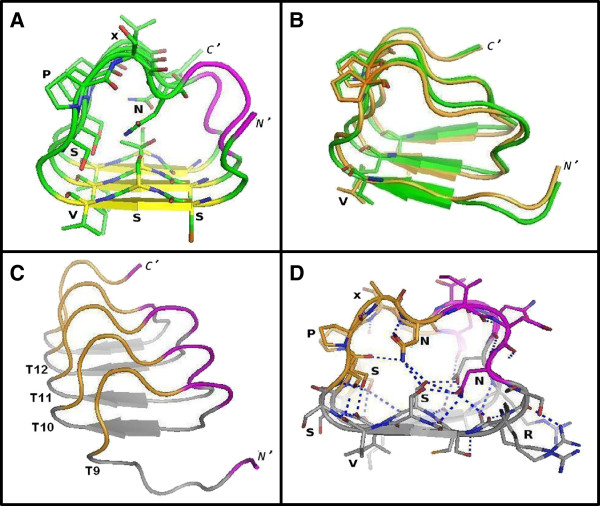
**3D-models of consecutive MR repeats. (A)** Initial model of three consecutive human MR repeats (T11 to T13, aa: 306-339). β-strands, turns and inter-repeat regions are colored in yellow, green and magenta, respectively. For clarity, only the side chains of residues discussed in the text are shown (stick models). The consensus sequence motifs, SSV and SPxN, are indicated. **(B)** Models resulting from the MD simulations. The dominant cluster of the last 50 ns of the REMD replica at 303 K and the most populated cluster of the last 30 ns of the solvated 50 ns MD simulation are shown in orange and green, respectively. **(C)** Final model (energy minimized) of five consecutive human MR repeats (T9 to T13, aa: 280-338) after the 20 ns MD simulation in explicit water. The β-turn structure corresponding to the SPxN motif is colored in orange. **(D)** Cross-section of the resulting β-helix core of the model shown in **C** (at repeats T10, T11). Similar residues forming ladders discussed in the text are labeled. Hydrogen bonds are indicated by dashed lines in blue. The molecular model illustrations of this figure were rendered using PyMOL.

In our initial 3D-model, each MR repeat was modeled as one coil of the β-helical structure, with the regions of each repeat corresponding to the consensus sequence motifs, SSV, at repeat positions 3 to 5 and SPxN, at positions 7 to 10 (Figure 6) modeled as the short β-strand and a β-like turn of each β-helical coil, respectively (Figure 7A and Additional file 1: Figure S1). The former was based on our CSSP prediction (Figure 3), whereas the latter was based on the observation that tetra-peptide SPxx motifs fold into compact β-turn-like structures [24]. As in the template structure, the short β-strands pack against each other to form a three-stranded β-sheet along the axis of the β-helix (Figure 7A and Additional file 1: Figure S1B). The core of the produced solenoid is formed exclusively by polar side-chains, mainly corresponding to inward-pointing serine and asparagine residues of the SSV and SPxN motifs (Figure 7A).

To test the stability of the produced β-helical model, we performed two types of independent molecular dynamics (MD) simulations. First, and in order to overcome kinetic trapping problems, one set of long enough (250 ns) replica-temperature exchange MD (REMD) simulations [25] was performed, at four temperatures (275, 303, 333 and 365 K) using implicit solvation. An additional, 50 ns long, classical MD simulation was carried out at a single temperature (300 K) with explicit treatment of water (TIP3P), to test the stability of the initial model in a more realistic environment. The dominant cluster of the last 50 ns of the REMD replica at 303 K (the closest to the physiological temperature) incorporated approximately 71% of the ensemble and corresponded to the initial β-helical fold (Figure 7B, in orange). The modeled β-helical structure remained also stable after the solvated 50 ns MD simulation (Figure 7B, in green), further supporting our β-helical model. Conservation of the β-helical fold and of the polar core was also observed in the dominant clusters of the REMD replicas at higher temperatures (data not shown), suggesting a high thermal stability of this particular β-helix. Indeed, burial of polar contacts has been shown to enhance the thermal stability of enzymes [26].

Next, two more coils, corresponding to two additional human MR repeats, were added to the initial structure to produce the 3D-model of five consecutive MR repeats (T9 to T13). The stability of this model was subsequently tested by a 20 ns classical MD simulation (at 300 K) with explicit treatment of water (TIP3P). The energy minimized model resulted from this MD simulation also showed preservation of the β-helical fold and of the polar core (Figures 7C, D). Monitoring of the secondary structure along the 20 ns MD trajectory (Additional file 1: Figure S2) demonstrated that the few secondary structure elements, namely the short β-sheet (in red) and the turn (in yellow) structures of the consensus SSV and SPxN motifs, remained rather stable during the entire MD simulation, with the exception of the N-terminal repeat (Additional file 1: Figure S2). This observation is in line with the notion that the repetitive units of solenoid proteins require one another to maintain structure [16]. Additional, transient β-strands, packed as an extra β-sheet perpendicular to the initial one, were formed at the inter-repeat T9-T10, T10-T11 and to a lesser extent, T11-T12, regions (marked with arrows in Additional file 1: Figure S2 and Additional file 1: Figure S3). The coordinates of this model in PDB format are available as Additional file 2: Table S2.

**Figure 8:**
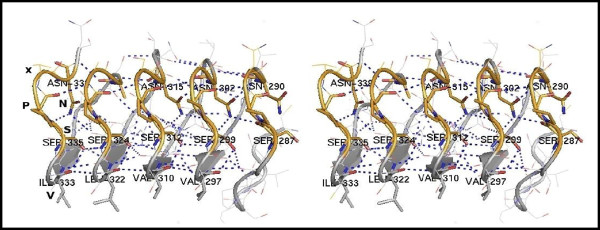
**Stereo view of details of the final model of the five consecutive human MR repeats (T9 to T13, aa: 280-338).** Conserved aliphatic, serine and asparagine residues forming ladders discussed in the text, are labeled and shown as stick models. Hydrogen bonds are indicated by dashed lines in blue. The figure was produced using PyMOL.

As predicted, during the course of the MD simulation, similar residues stacked against each other (Figure 7D), stabilizing the β-helical structure through hydrogen bonding along the axis of the β-helix (Figure 8). In particular, the side chains of the inward-pointing serines and asparagines of the conserved SPxN motif of each repeat, imposed by its β-like turn structure (Figures 7D), hydrogen bonded to main-chain carbonyl oxygens and amide nitrogens of the same and preceding β-helical coils, leading to the formation of extensive serine and asparagine ladders, respectively (Figure 8). Such interior, polar stacks have been proposed to stabilize turns in β-helical folds [18,27]. Indeed, the turn structure of the SPxN motif of each MR repeat remained remarkably stable during the entire solvated 20 ns MD simulation (Additional file 1: Figure S2, in yellow). Furthermore, hydrogen bonded ladders of internal polar residues at both conserved and variant positions, reinforced the polar core of the resulting β-helix (Figure 7D).

In its turn, the stacking of the SPxN turns, through extensive hydrogen bonding along the β-helical axis, caused a remarkable stacking of the conserved prolines of this motif (Figure 8). Proline residues, although uncommon, have been also found in other β-helical proteins (pectin methyltransferase and the receptor for insulin-like growth factor, IGFR1) (See [18] and references therein). The proline stacking predicted here for the MR repeats and a similar stacking of Pro 46 and Pro 71 in IGF1R, demonstrate that proline residues can be accommodated in β-helices and “extend our idea of what can be efficiently stacked”, as also suggested by Jenkins *et al.*[18].

The packing of polar residues into the interior of the modeled β-helix forced the few conserved aliphatic residues, at repeat position 5 (Figure 6), to adopt a solvent-exposed orientation in our initial model (Figure 7A). During the course of the MD simulations, these residues stacked (Figures 7B and D) to form an external hydrophobic stripe (Figure 8), implying a role for this surface as an interacting platform and/or as a dimerization domain. Indeed, stacking of solvent-exposed hydrophobic residues has been observed in several β-solenoids and has been mainly linked to homo-oligomerization ([15] and references therein). The conservation of the hydrophobic character of this repeat position (position 5 in Figure 6), in conjunction with the observation that the folding of the MR-MD repeat fragment is stabilized in the presence of TFE [14], which mimics a partial hydrophobic environment, further support our model. Furthermore, some conserved cysteine residues occupy the adjacent semi-variant repeat position 6 (Figure 6), which according to our model is also solvent-exposed (Additional file 1: Figure S1B; Right) and may thus contribute to inter-molecular interactions or dimerization through the formation of inter-molecular Cys-Cys bonds. In addition, basic residues corresponding to MR repeat position 2 (Figure 6) also stacked during the course of the 20 ns MD simulation, forming an exposed basic surface (Figure 7D), which could also serve as a molecular recognition platform, reinforcing our idea of the MR repeat β-helical fold acting as a protein interaction and/or dimerization scaffold.

Taken together, our observations strongly support a β-helical structure for the repeat region of the MR inhibitory domain.

## Discussion

We have described a novel repeat specific to the MR, present in bony fishes and tetrapoda, which in the human MR conforms to an ensemble of at least 15 repeats extending for about 200 aa, forming most of the inhibitory domain (ID) of the MR. This repeat is defined by a 10 aa pattern with a conserved Ser-Pro motif at positions 7 and 8. The spacers between the repeats are often of 2 or 3 aa, indicating that it forms a very compact structure.

This domain of tandem repeats in the MR seems to have evolved by tandem repeat duplication in “just” over 50 million years to a number of units that became fixed for the following 400 million years (since the establishment of the tetrapoda lineage). We could detect only one other event of repeat duplication, which happened in bony fishes and evidenced an ancestral situation where there was evolutionary pressure to increase the size of the ensemble by repeat duplication. Tandem repeat structures can easily accommodate such duplications since the packing of consecutive units is not affected by the insertion of a new one, as far as the periodicity is maintained [5]. The fact that in tetrapoda the number of units remained constant for 400 million years suggest that the length of the domain of repeats is of functional importance.

Based on several lines of evidence (periodicity, secondary structure prediction, solenoid-prediction, previously published CD data, etc.) and using comparative molecular modeling in combination with molecular dynamics simulations, we predicted here that consecutive MR repeats are compatible with a β-solenoid fold, namely a β-helical structure. Solenoid structures often serve protein-protein interactions [5] and appear to promote dimerization (or other homo-oligomerization) of multidomain proteins [15]. Since the MR is also involved in many inter- and intra-molecular interactions [28], including homo-and hetero-dimerization [29], we hypothesize that the function of the repeat ensemble within the MR inhibitory domain is to serve some of these interactions by presenting various interacting surfaces through the formation of a β-solenoid fold.

Indeed, according to our 3D-model, several polar residues at conserved repeat positions stack in the interior of the β-solenoid stabilizing the β-helical fold, whereas hydrophobic as well as basic residues cluster on the surface of the β-helix implying an important role of this fold and of the resulting surfaces as interacting platforms. In particular, the β-solenoid surface resulting from the stacking of the solvent-exposed conserved apolar residues at MR-repeat position 5 (Figures 6, 7D and 8), may promote dimerization (as also observed in several other β-solenoid proteins [15]) and intra- or inter-molecular hydrophobic interactions of MR.

**Figure 9:**
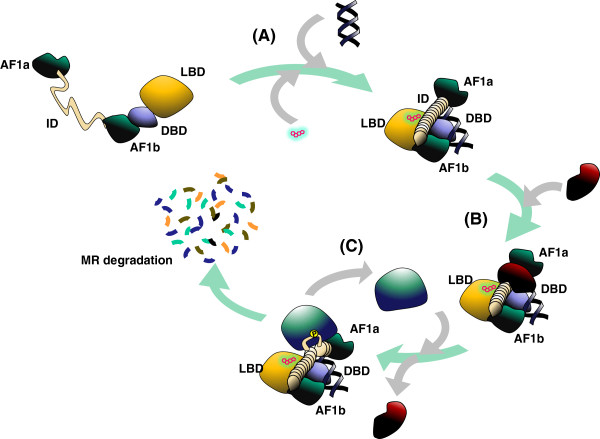
**Cartoon summarizing our findings. (A)** The structure of the MR ID could be stabilized in response to DNA binding. **(B)** In its folded state, the ID remains available as a scaffold for protein interactions. **(C)** Specific phosphorylation of buried serines requires opening of the ID structure and leads to degradation of the MR.

A study of a fragment of the ID (named MR middle domain, MR-MD, residues 247 to 365), missing repeats 1 to 6 (see Figure 1), demonstrated that binding of several transcriptional co-regulatory proteins, acting either as co-repressors (SMRT and Rip140) or co-activators (SRC2, SRC3 and CBP) required prior folding of this fragment [14]. On the other hand, this fragment does not seem to possess a stable structure, neither in isolation nor in the context of the full NTD, as demonstrated by CD experiments on the MR-MD and MR-NTD domains in the absence of structure stabilizers [14]. Instead, the β-helical fold of the complete repeat region may be stabilized in the context of the full receptor e.g. via an inter-domain allosteric mechanism such as the allosteric interaction between the NTD and DBD domain in response to DNA binding, as proposed for other steroid receptors (see [30] and [31] and references therein). Acquisition of an ordered conformation, through a “cross-talk” between the NTD and an extended DBD-containing fragment of human GR has, in turn, been correlated with the interaction of the GR-NTD with co-regulatory proteins [31]. It is tempting to speculate that a similar “cross-talk” exists between the MR-NTD and the MR-DBD-hinge regions in response to DNA binding, which in turn stabilizes the β-helical fold of the repeat region allowing interactions with co-regulatory proteins. In line with this hypothesis, the MR-NTD-DBD fragment has been shown to be able to exert trans-repression activity on a reporter gene in the presence of the SMRT co-repressor, whereas this activity was abrogated when a deletion mutant, lacking the MR-MD region, was used instead [14]. Taken together, these observations strongly support the idea of a stabilized β-helical fold of the MR-repeat region in response to DNA binding, serving as interaction platform for various co-repressor proteins, thus contributing to the action of this region as a transcriptional inhibitory domain (Figure 9).

β-solenoids have been predicted to be located immediately before and/or after functional domains [32] and the MR-repeat region is flanked by the MR AF1a and AF1b trans-activation domains. Thus, in addition to providing interaction surfaces for co-repressor proteins, the proposed β-solenoid fold of the MR-repeat region may also play an indirect role in the function of MR by e.g., regulating (controlling) the relative positioning of its transcriptional trans-activation domains. Indeed, both the AF1a and AF1b regions have been shown to be involved in the aldosterone-specific MR inter-domain interaction between the NTD and LBD (N/C interaction), which in addition, has been proposed to be influenced by the distance between the two interacting domains [33]. In support to this idea, the N/C interaction in MR was found to be specific to its N-terminus as substitution by the GR or AR N-terminus, lacking the repeat region, did not allow interaction with the MR LBD (see [34] and references therein).

Phosphorylation of steroid receptors has been shown to play an important role in modulating their function and most of the sites identified after hormone-treatment, include Ser-Pro motifs located at their NTDs ([35] and references therein). The MR contains multiple phosphorylation sites regulated by different kinases ([36] and references therein), including phosphorylation of the serines of many of the SPxN motifs of the repeats such as Ser299 [8], from repeat T10, and serines 196, 227, 238, 263, 287 and 361 (corresponding to the consensus ERK-related phosphorylation motif, X-P-X(1-3)-SP-X at repeats T2, T4, T5, T7, T9 and T15, respectively; Figure 2) that were recently reported to undergo rapid aldosterone-induced phosphorylation by the ERK1/2 kinase [9]. According to our 3D-model, such serines appear to be inward-pointing and therefore inaccessible to kinases in the proposed folded form of the repeats (for example, see Ser299 in Figure 8); their phosphorylation would require a prior opening (unfolding) of the β-helical structure, at least locally. This could be achieved, for example, by another type of phosphorylation at exposed site(s). Alternatively, unfolding prior to phosphorylation may be induced by the kinase itself. Unfolding of their substrates upon docking and prior to phosphorylation has been attributed to some kinases working through docking motifs ([37] and references therein) including ERK2 [38]. The observed increase of the sedimentation coefficient of Ser/Thr-phosphorylated MR (from 5.1S to 8.8S) [39], suggestive of a (Ser/Thr)phospho-induced opening of the MR structure, and the fact that many phosphorylation sites exist in the MR [36], support the idea of a specific phospho-induced unfolding of the helical structure of the MR inhibitory domain (Figure 9). In further support to this idea, aldosterone-induced sequential phosphorylation of MR repeats via ERK1/2 results in destabilization of the receptor through a polyubiquitylation/degradation mechanism [9]. Furthermore, the observation that this specific phosphorylation by ERK1/2 disrupts the Tsg101/MR interaction leading to monoubiquitin removal from MR [9], implies an important role of the proposed β-helical structure of these MR repeats in Tsg101 binding and in preserving the monoubiquitylation state of the receptor. Based on these observations, it is tempting to speculate that the β-helical fold may offer an additional mechanism to prevent unspecific phosphorylation of MR.

## Conclusions

In conclusion, we propose that the inhibitory domain of the MR contains sequence repeats compatible with a β-helical fold offering a scaffold for multiple intra-and inter-molecular interactions (including dimerization) and that these interactions are modulated via conformational changes, involving β-helix to random transitions, regulated by specific kinases, thus playing an important role in the coordination and sequential interactions of various MR partners and therefore in the specificity and in the (patho)physiological function of this receptor. We expect that these results should guide future research on the mechanisms of MR function.

## Methods

### Sequence similarity searches

Initial scan for homologs of the human MR was done using the NCBI BLAST server against the NCBI’s non-redundant protein sequence database with default parameters [40].

For a HMM profile search of homologs with MR repeats, we first computed a multiple sequence alignment of all possible pairs of consecutive repeats of the human and *Danio rerio* MR sequences reported in Figure 2. We used this alignment to scan the UniProtKB database of protein sequences using the hmmsearch option of the HMMER web server with default parameters [11]. This search did not report significant hits other than MR proteins.

### Multiple sequence alignment

For the graphical display of the MR repeat sequences in human MR and its homologs, we compiled a multiple sequence alignment of the human and *Danio rerio* MR sequences with MR sequences from selected species from tetrapoda and another bony fish (Figure 2). For simplicity, this alignment was also used for the analysis of identity levels between each of the human repeats and repeats in other tetrapoda shown in Figure 4. There are many other MR sequences in the protein sequence databases that were not used in the analysis.

### Regular expression for detection of repeats

For an initial definition of a region of tandem repeats in a multiple sequence alignment of human MR and homologs, matches to regular expressions of increasing complexity were visualized with jalview [7]. Using an *ad hoc* procedure, we started detecting the motif SP, and then a regular expression was step-wise increased in size and types of amino acids accepted, attempting to match as many hits in the region contiguous to the first repeats identified and as few hits as possible in other distant parts of the sequences.

### Secondary structure predictions

Secondary structure prediction was carried out using the contact-dependent secondary structure propensities (CSSP) [13] tool (available at http://cssp2.sookmyung.ac.kr) and the MR repeat sequence as query.

### Solenoid predictions

Solenoid predictions were performed using the REPETITA algorithm presented in [17] using the REPETITA web tool (http://protein.bio.unipd.it/repetita) and the sequence of the MR repeats region as query.

### Construction of initial 3D-models

The 3D-model of three consecutive hMR repeats (T11 to T13; aa: 306-339) was constructed as a three-coiled right-handed parallel β-helix, using the crystal structure of the *T. molitor* antifreeze protein (PBD code: 1EZG) [23], as template. The program Swiss-PdbViewer [41] and a manual editing of the sequence alignment between MR repeats and the template were used for this purpose. The sequence alignment was dictated by the following rules: (i) according to contact-dependent secondary structure predictions and the observation that most SPxx tetra-peptides fold into compact β-turn-like structures [24], the regions complying with the consensus sequence motifs, SSV and SPxN of each MR repeat should correspond to the β-strand and a β-like-turn of the β-helical coils of the template structure, respectively, and (ii) sites of insertions/deletions in the aligned repeats should be in loops. Inter-repeat loops were modeled using the *build-loop* utility of the Swiss-PdbViewer program. Ace-, Nme-blocked termini were added to the model to minimize the possibility of salt-bridge traps resulting from the charged termini. This model was subsequently used as the starting conformation for a set of replica-temperature exchange molecular dynamics (MD) simulations and a classical, 50 ns long, MD simulation in explicit water (see below).

The 3D-model of five consecutive hMR repeats (T9 to T13, aa: 280-338) was constructed as a five-coiled right-handed parallel β-helix by adding two additional β-helical coils to the most populated cluster (see below) of the 50 ns classical MD simulation of the three-repeat model, using the same rules and program, as described above. This model was subsequently used as the starting conformation for the 20 ns long MD simulation in explicit water (see below).

### Molecular dynamics simulations

Molecular Dynamics (MD) simulations were performed using the GROMACS4 (v. 4.5.3) software package [42] through an updated version of the Gromita GUI that we developed recently [43]. The improved version of the AMBER99-SB force field, AMBER99SB-ILDN [44], as implemented in GROMACS4, a time step for integration of the potential function of 2 fs and the LINCS algorithm for covalent bonds [45] were used in all MD simulations.

#### Replica exchange molecular dynamics

Replica-temperature Exchange MD (REMD) simulations [25] were performed starting from the β-helical conformation of three consecutive MR repeats, modeled as described above. Four replicas were used with temperatures of 275, 303, 333 and 365 K, respectively. 250 ns were performed for each replica, and a replica exchange was attempted every 1000 MD steps. Simulations were carried out using implicit solvation (GB/SA) and the OBC (II) model [46] for calculating Born radii. A cutoff of 10 Å was used for non-bonded interactions.

#### MD simulations in explicit water

The MD simulations in explicit water were carried out using periodic dodecahedron boxes filled with 1,624 and 3,100 TIP3P water molecules [47] to solvate the MR three- and five-repeat models, respectively. Periodic boundaries were applied to minimize edge effects. The systems were neutralized with counter-ions. The solvated systems were first optimized by conjugate gradient energy minimization combined with a steepest descent minimization performed every 100 steps. Subsequently, the systems were subjected to restrained MD simulations of 100 ps at 300 K, where the protein atoms were harmonically restrained to their initial position with a force constant of 1,000 kJ mol^-1^ nm^-2^ to allow the solvent to equilibrate. The optimization phase was followed by 50 and 20 ns of unrestrained MD simulations at 300 K, for the three- and five-repeat models, respectively. The NVT ensemble was used and the overall temperature was kept constant, coupling protein and solvent separately at 300 K using velocity rescaling [48]. The v-rescaling method was preferred over the commonly used Berendsen thermostat, because it has been shown to give a better distribution of the kinetic energy [48]. The long-range electrostatic interactions were evaluated using the particle mesh Ewald method [49] with a grid size of less than 0.12 nm. A non-bonded cutoff of 8 Å was used for both MD simulations. Rigid water using the SETTLE algorithm [50], was used in this type of simulations.

The five-repeat 3D-model obtained after the 20 ns MD simulation in explicit water was subsequently optimized using 50,000 steps of conjugate gradient energy minimization with flexible water.

### Analysis of the MD trajectories

Analysis of the MD trajectories was focused on monitoring the secondary structure during the MD simulations using the DSSP criteria [51] through the *do_dssp* module of GROMACS. Cluster analysis used the *g_cluster* module of GROMACS. The VMD program [52] was used for the visualization of the trajectories and molecular model illustrations were rendered using PyMOL and VMD.

## Competing interests

The authors declare that they have no competing interests.

## Authors’ contributions

MV carried out the structural analyses, KB and MAA carried out the sequence and phylogenetic analyses, MV and MAA wrote the manuscript, all authors read and approved the final manuscript.

## Supplementary Material

Additional file 1: Table S1 Repeats detected in the human MR using web tools. **Figure S1.** 3D-modeling of the MR repeats. **Figure S2.** Analysis of the 20 ns MD trajectory of the five consecutive MR repeats. **Figure S3.** One snapshot (at 19.5 ns) of the solvated 20 ns MD simulation of the five consecutive human MR repeats (T9 to T13). Click here for file

Additional file 2: Table S2 Contains the coordinates of the final 3D-model of repeats T9 to T13 of human MR (aa: 280-338). Click here for file
