# Supplementary material for: Short tandem repeats in the inhibitory domain of the mineralocorticoid receptor: prediction of a β-solenoid structure
Source: BMC Struct Biol. 2013 Oct 2;13:17. doi: 10.1186/1472-6807-13-17 (PMC3851330; doi:10.1186/1472-6807-13-17)
Supplement: Additional file 1: Table S1 — Repeats detected in the human MR using web tools. Figure S1. 3D-modeling of the MR repeats. Figure S2. Analysis of the 20 ns MD trajectory of the five consecutive MR repeats. Figure S3. One snapshot (at 19.5 ns) of the solvated 20 ns MD simulation of the five consecutive human MR repeats (T9 to T13). [file 1472-6807-13-17-S1.doc]

**SUPPLEMENTARY MATERIAL FOR:**

Short tandem repeats in the inhibitory domain of the mineralocorticoid receptor: prediction of a β-solenoid structure.

Metaxia Vlassi*^1^, Katharina Brauns ^2^, Miguel A. Andrade-Navarro*^2^

^1^ Protein Structure & Molecular Modeling laboratory, Institute of Biosciences & Applications, National Centre for Scientific Research “Demokritos”, 15310 Ag. Paraskevi, Athens, Greece

^2^ Max Delbrück Center for Molecular Medicine, Robert-Rössle-Str. 10, 13125 Berlin, Germany

*Corresponding authors

**Supplementary tables**

**Supplementary Table S**1. Repeats detected in the human MR using web tools

| Method^1^ | Region | Sequences | Length | Ref |
| --- | --- | --- | --- | --- |
| Hhrep | 266-348 | SSMKSSISSPPS....  HCSVKSPVS---spnn  VTLRSSVSSPA-ni..  NNSRCSVSSPSN....  TNNRSTLSSPAA....  STVG-SICSPVN....  NAFSYTASGT--.... | 10 |  |
| RADAR | 155-353 | ..SCVNTPL...RSF.M.SD...SGSSVNGG  MRAVVKSPI[9]CSP.L..N...MTSSVCSP  HASNVGSPL...SSP.L.SS...MKSSISSP  ...SVKSPV...SSP.NnVT...LRSSVSSP  NINNSRCSV...SSPsN.TN...NRSTLSSP  A.STVGS.I...CSP.V.NN[6]SGTSAGSS | 20 |  |
| TRUST | 189-339 | EKSPSVCSPLN--  -MTSSVCSPAGIN  TLRSSVSSPANIN  NSRCSVSSPSNTN  STVGSICSPVN-- | 12 |  |
| T-REKS | 262-276 | SSPL-  SS-MK  SS-I-  SSPP- | 4 |  |

^1^We also used XSTREAM , which did not find repeats.

**Supplementary figures**

**Supplementary Figure S1.** **3D-modeling of the MR repeats.** Each MR repeat was modeled as one coil of a right-handed β-helix using the crystal structure of *T. molitor* antifreeze protein (PDB code:1EZG, ) as template with the region of each MR repeat corresponding to the consensus sequence motifs, SSV (repeat positions 3 to 5) and SPxN (positions 7 to 10) modeled as the short β-strand (based on our CSSP prediction) and a β-like turn (based on the observation that tetra-peptide SPxx motifs fold into compact β-turn-like structures ) of each β-helical coil of the template structure, respectively. (A) Alignment of consensus repeat sequences used to guide the 3D-modeling procedure. The MR consensus sequence is derived from the sequence logo shown in Figure 6. “x” denotes any amino-acid. The numbering of the MR sequence refers to repeat positions. The arrows and the blue bar indicate the position of modeled and CSSP predicted β-strands, respectively. This figure was produced using ESPript . (B) *Left*: Three β-helical coils corresponding to three consecutive repeats (aa: 36-71) of the crystal structure (1EZG) used as template in the modeling procedure. *Right*: Initial 3D-model of three β-helical coils corresponding to repeats T11 to T13 of human MR (aa: 305-338). Structure representation and coloring is as in Figure 7A. Labels correspond to consensus repeat sequences. The molecular model illustrations of B were rendered using PYMOL.

**Supplementary Figure S2.** **Analysis of the 20 ns MD trajectory of the five consecutive MR repeats.** Monitoring of the secondary structure of the modeled T9 to T13 MR repeats over the course of the solvated 20 ns MD simulation. The sequence of each modeled repeat is indicated. Conserved aliphatic residues of the SSV motif are boxed and residues corresponding to the SPxN motif are shown in red. The secondary structure elements are colored as shown at the bottom of the figure. With the exception of the N-terminal repeat, the β-sheet and turn structures (in red and yellow, respectively) remained rather stable during the entire simulation, in line with the notion that the repetitive units of solenoid proteins require one another to maintain structure .

**Supplementary Figure S3.** **One snapshot (at 19.5 ns) of the solvated 20 ns MD simulation of the five consecutive human MR repeats (T9 to T13).** The coloring of secondary structure elements is as in Figure 7A. The additional, transient β-sheet at the inter-repeat regions, T9-T10 and T10-T11, is indicated with an arrow. This figure was created using the VMD program .

**References**

1. Biegert A, Soding J: **De novo identification of highly diverged protein repeats by probabilistic consistency**. *Bioinformatics* 2008, **24**(6):807-814.

2. Heger A, Holm L: **Rapid automatic detection and alignment of repeats in protein sequences**. *Proteins* 2000, **41**(2):224-237.

3. Szklarczyk R, Heringa J: **Tracking repeats using significance and transitivity**. *Bioinformatics* 2004, **20 Suppl 1**:i311-317.

4. Jorda J, Kajava AV: **T-REKS: identification of Tandem REpeats in sequences with a K-meanS based algorithm**. *Bioinformatics* 2009, **25**(20):2632-2638.

5. Newman AM, Cooper JB: **XSTREAM: a practical algorithm for identification and architecture modeling of tandem repeats in protein sequences**. *BMC Bioinformatics* 2007, **8**:382.

6. Liou YC, Tocilj A, Davies PL, Jia Z: **Mimicry of ice structure by surface hydroxyls and water of a beta-helix antifreeze protein**. *Nature* 2000, **406**(6793):322-324.

7. Suzuki M, Yagi N: **Structure of the SPXX motif**. *Proc Biol Sci* 1991, **246**(1317):231-235.

8. Gouet P, Courcelle E, Stuart DI, Metoz F: **ESPript: analysis of multiple sequence alignments in PostScript**. *Bioinformatics* 1999, **15**(4):305-308.

9. Kajava AV: **Tandem repeats in proteins: from sequence to structure**. *J Struct Biol* 2012, **179**(3):279-288.

10. Humphrey W, Dalke A, Schulten K: **VMD: visual molecular dynamics**. *J Mol Graph* 1996, **14**(1):33-38, 27-38.
